# Effect Modification of Rescue Stenting in ICAS-Related Middle Cerebral Artery Occlusion: The Role of Thrombectomy Failure, Stenosis Severity, and ASPECTS

**DOI:** 10.1007/s12975-026-01496-9

**Published:** 2026-09-23

**Authors:** Andrea M. Alexandre, Luca Scarcia, Adam A. Dmytriw, Valerio Brunetti, Arturo Consoli, Wen Sun, Yingjie Xu, Xianjun Huang, Joseph D. Gabrieli, Charlotte Chung, Alessandro Sgreccia, Mohamad Abdalkader, Yan-Lin Li, Shruthika Kimavath, Mohd Shariq, Giovanni Frisullo, Nicola Limbucci, Francesco Arba, Alessandro Pedicelli, Mauro Gentile, Serena Abruzzese, Mariangela Piano, Maria P. Ganimede, Emilio Lozupone, Chiara Gaudino, Riccardo Russo, Julien Burel, Julien Allard, Nicolas Chausson, Roberta Partesano, Nicola Cavasin, Nicolò Mandruzzato, Pietro Trombatore, Antonio A. Caragliano, Federico Mazzacane, Giancarlo Salsano, Antioco Sanna, Pietro Panni, Andrea Zini, Frédéric Clarençon, Eytan Raz, Thanh N. Nguyen, Aldobrando Broccolini

**Affiliations:** 1 Interventional Neuroradiology Unit, Fondazione Policlinico Universitario A. Gemelli IRCCS, Rome, Italy; 2 Department of Neuroradiology, Henri Mondor Hospital, Creteil, France; 3 Nuffield Department of Surgical Sciences, Medical Sciences Division, University of Oxford, Oxford, UK; 4 Queens Hospital, Institute of Therapeutic Neuroradiology, Barking, Havering and Redbridge University Hospitals NHS Trust, Romford, UK; 5 Neurology Unit, Fondazione Policlinico Universitario A. Gemelli IRCCS, Rome, Italy; 6 Department of Diagnostic and Therapeutic Neuroradiology, Hôpital Foch, Suresnes, France; 7 Department of Neurology, Centre for Leading Medicine and Advanced Technologies of IHM, The First Affiliated Hospital of USTC, Hefei, Anhui China; 8 Department of Neurology, Yijishan Hospital, Wannan Medical College, Wuhu, Anhui China; 9 Neuroradiology Unit, Padua University Hospital, Padua, Italy; 10 Department of Neurosurgery and Department of Radiology, NYU Langone Health, New York, NY USA; 11 Department of Radiology and Neurology, Boston Medical Center, Boston, MA USA; 12 Interventional Neurovascular Unit, Azienda Ospedaliero-Universitaria Careggi, Firenze, Italy; 13 Stroke Unit, Azienda Ospedaliero-Universitaria Careggi, Firenze, Italy; 14 Department of Neurology and Stroke Center, IRCCS Istituto delle Scienze Neurologiche di Bologna, Maggiore Hospital, Bologna, Italy; 15 Neuroradiology Unit, ASST Grande Ospedale Metropolitano Niguarda, Milan, Italy; 16 Neuroradiology Unit, Presidio Ospedaliero Centrale SS Annunziata, Taranto, Italy; 17 Neuroradiology Unit, Hospital Vito Fazzi, Lecce, Italy; 18 Department of Neuroradiology, Azienda Ospedaliero-Universitaria Policlinico Umberto I, Rome, Italy; 19 Neuroradiology Unit, Azienda Ospedaliero-Universitaria Città della Salute e della Scienza, Turin, Italy; 20 Department of Radiology, Rouen University Hospital, Rouen, France; 21 Department of Interventional Neuroradiology, Pitié-Salpêtrière University Hospital, Paris, France; 22 Department of Neurology, Centre Hospitalier Sud Francilien, Corbeil-Essonnes, France; 23 Neuroradiology Unit, Azienda Ospedaliero Universitaria di Parma, Parma, Italy; 24 Neuroradiology Unit, Ospedale dell’Angelo, Mestre, Italy; 25 Interventional Neuroradiology, Azienda Ospedaliera Universitaria Integrata di Verona, Verona, Italy; 26 Department of Diagnostic Imaging and of Interventional Radiology and Neuroradiology, Azienda Ospedaliera Garibaldi, Catania, Italy; 27 Neuroradiology Unit, Azienda Ospedaliera Universitaria Policlinico G. Martino, Messina, Italy; 28 Department of Stroke Unit and Emergency Neurology, IRCCS Mondino Foundation, Pavia, Italy; 29 Neuroradiology Unit, IRCCS Ospedale Policlinico San Martino, Genova, Italy; 30 Neuroradiology Unit, Santa Corona Hospital, Pietra Ligure, Italy; 31 Interventional Neuroradiology Unit, IRCCS San Raffaele University Hospital, Milan, Italy; 32 Department of Neuroscience, Catholic University School of Medicine, Rome, Italy

**Keywords:** Intracranial artery stenosis, Mechanical thrombectomy, Rescue stenting, Large vessel occlusion, ASPECTS, Treatment-effect heterogeneity

## Abstract

Rescue stenting (RS) is increasingly used for intracranial artery stenosis-related large vessel occlusion (ICAS-LVO), but optimal patient selection remains uncertain. We investigated the baseline and procedural factors associated with receiving RS and whether they modified the association between RS and clinical outcome.

This retrospective multicenter study included patients with middle cerebral artery (MCA) ICAS-LVO treated with mechanical thrombectomy (MT) alone or MT followed by RS. Baseline and procedural characteristics associated with receiving RS were identified using multivariable logistic regression and incorporated into inverse probability of treatment weighting models to assess treatment-effect heterogeneity for the 90-day modified Rankin Scale score.

Among 264 patients, 193 received RS and 71 MT alone. MT failure, severe stenosis (> 75%), and higher baseline Alberta Stroke Program Early CT Score (ASPECTS) were independently associated with receiving RS. Both MT failure (interaction OR 0.28, 95% CI 0.10–0.83; p for interaction = 0.02) and severe stenosis (interaction OR 0.13, 95% CI 0.03–0.53; p for interaction = 0.004) significantly modified the association between RS and clinical outcome, whereas baseline ASPECTS did not. Within the MT failure subgroup, a significant interaction between severe stenosis and ASPECTS was observed (interaction OR 0.08, 95% CI 0.02–0.38; p for interaction = 0.002), suggesting a more favorable association between RS and outcome at higher ASPECTS values in patients with severe stenosis.

In MCA ICAS-LVO, RS may be associated with more favorable outcomes in patients with MT failure, particularly in the presence of severe residual stenosis and preserved ASPECTS. These findings support a more selective approach to RS and warrant prospective validation.

**Supplementary Information:**

The online version contains supplementary material available at https://doi.org/10.1007/s12975-026-01496-9.

## Introduction

Mechanical thrombectomy (MT) is the standard of care for acute large vessel occlusion (LVO), achieving higher recanalization rates than medical therapy alone [1]. However, MT fails in 10–20% of patients, often due to challenging vascular anatomy, resistant or calcified thrombus, or underlying intracranial artery stenosis (ICAS) [2–4]. Compared with embolic occlusions, ICAS-LVO is associated with lower rates of durable recanalization and higher risk of re-occlusion, which may contribute to worse functional outcomes [2].

Rescue therapies, including intracranial angioplasty and stenting, have increasingly been adopted to restore and maintain vessel patency in ICAS-LVO after failed or unstable thrombectomy. Although observational studies and multicenter registries have generally suggested improved angiographic and clinical outcomes with rescue stenting (RS), particularly in MT-refractory occlusions, the interpretation of these findings remains challenging because treatment allocation is highly selective and influenced by intraprocedural findings [5–11]. Moreover, the benefit of RS in patients achieving recanalization despite residual stenosis remains uncertain, and randomized evidence is limited [8]. Notably, a recent randomized study failed to demonstrate a clear overall benefit of rescue therapies over medical management alone in patients with unsuccessful recanalization or severe residual stenosis after MT [12].

As a result, the optimal management of acute ICAS-LVO remains uncertain, and substantial variability in practice persists among neurointerventionalists, reflecting both the lack of standardized protocols and ongoing concerns about procedural risks, including hemorrhagic complications and stent thrombosis [13–15]. Decision-making is therefore largely individualized, and factors involved in real-world treatment allocation may also modify the association between RS and clinical outcome, raising the possibility of treatment-effect heterogeneity across patient subgroups.

The aims of the present retrospective study were (1) to identify baseline and procedural variables independently associated with the decision to perform emergent RS in patients with middle cerebral artery (MCA) ICAS-LVO, thereby characterizing the real-world treatment allocation, and (2) to explore whether these same treatment-allocation variables modified the association between RS and clinical outcome, thus identifying potential treatment-effect heterogeneity.

## Methods

This retrospective observational study involved 28 comprehensive stroke centers across Europe, the United States, and China and was conducted within the framework of a non-profit study protocol approved by the Ethics Committee of the Coordinator Center (protocol number 6410/20, ID 3004), in accordance with the Declaration of Helsinki. The local Ethics Committee approved the use of the patient data. The need for informed consent was waived due to the retrospective nature of the study and because all therapeutic procedures were routine care. The analysis was conducted in adherence with the STrengthening the Reporting of OBservational studies in Epidemiology statement [16].

The prospective databases of the participating centers were screened for consecutive patients with acute MCA occlusion who underwent endovascular treatment between January 2020 and June 2024. Mechanical thrombectomy was performed with a stent-retriever, direct contact aspiration, or a combined technique. Only patients with underlying ICAS in the M1 or proximal M2 segment of the MCA identified during MT were included in the study. Intracranial artery stenosis was defined as a 50–99% residual fixed stenosis or immediate re-occlusion not attributable to alternative causes such as vessel dissection or vasospasm, in accordance with previously published criteria [5, 8]. The degree of MCA stenosis was measured on post-thrombectomy angiograms using a method adapted from the Warfarin-Aspirin Symptomatic Intracranial Disease (WASID) trial, as follows: [1-(D-stenosis/D-normal)]⋅100, where D-stenosis is the diameter of the artery at the site of the most severe stenosis and D-normal is the diameter of the proximal normal artery [17, 18]. For analytical purposes, stenosis severity was categorized into moderate (50–75%) and severe (> 75%). The decision to perform RS, with or without angioplasty, after MT was at the discretion of the neurointerventionalist or based on local protocols. Radiological and angiographic data were reviewed locally by neuroradiologists/neurointerventionalists blinded to clinical information. Patients were divided into two groups based on whether they received MT only or MT followed by RS.

### Measures of Outcome

Receiving RS was the outcome measure of the first aim of the study. For the second aim, the primary outcome measure was the ordinal modified Rankin Scale (mRS) score, acquired 90 days after stroke. Efficacy and safety outcome measures included: (1) successful recanalization at the end of the endovascular procedure, defined as final modified Treatment in Cerebral Infarction (mTICI) grade ≥2b, and complete/near-complete recanalization, defined as final mTICI grade 2c-3; (2) occurrence of symptomatic intracranial hemorrhage (sICH), defined as any parenchymal hematoma associated with an increase of ≥ 4 points in the National Institutes of Health Stroke Scale (NIHSS) score or ≥ 2 points in any NIHSS subcategory, according to the Heidelberg bleeding classification [19]; and (3) 90-day mortality.

### Statistical Analysis

Baseline characteristics were summarized using descriptive statistics. Continuous variables were reported as medians and interquartile ranges (IQR), and categorical variables as counts and percentages. Between-group differences were assessed using Fisher’s exact test for categorical variables and the Mann-Whitney U test for continuous variables.

The analysis was structured in two stages. First, variables associated with the real-world decision to perform RS were identified using stepwise logistic regression. Candidate variables included cardiovascular risk factors, baseline clinical and radiological characteristics, and procedural factors. Variables with *p* < 0.10 in univariate analysis were entered into a multivariable logistic regression model, followed by stepwise backward selection. Odds ratios (ORs), 95% confidence intervals (CIs), and p-values were calculated. Model discrimination was assessed using the area under the receiver operating characteristic curve (AUC), and calibration was evaluated using the Hosmer-Lemeshow test. Second, the variables independently associated with RS in this multivariable model were used to estimate each patient’s propensity score, defined as the probability of receiving RS. This approach was selected because the objective was to determine whether the same factors that characterized real-world treatment allocation also modified the association between RS and outcome. Stabilized weights were computed to balance the treatment-allocation variables between groups, and covariate balance was assessed using standardized mean differences (SMDs), with values < 0.1 indicating acceptable balance. Propensity score overlap and effective sample size diagnostics were also evaluated to assess the stability of the weighted estimates. Inverse probability of treatment weighting (IPTW)-adjusted ordinal logistic regression was used to evaluate the association between RS and the 90-day mRS score, whereas logistic regression was used for binary outcomes. Exploratory interaction terms between RS and each treatment-allocation variable were included in IPTW-weighted models to assess whether the association between RS and outcome differed across subgroups. For ordinal outcomes, interaction ORs quantified differences between the subgroup-specific common ORs for RS, with values < 1 indicating a more favorable association between RS and a shift toward lower 90-day mRS scores. For binary outcomes, interaction ORs quantified differences in the association between RS and each outcome across subgroups. The 95% CIs and p-values for interaction terms were calculated accounting for the weighting design. As a sensitivity analysis, the primary IPTW-weighted interaction models were additionally adjusted for clinically relevant prognostic variables not included in the propensity score model.

All analyses were performed using R software v.4.3.2 (R Foundation for Statistical Computing, Vienna, Austria), with the *tidyverse*, *WeightIt*, *survey*, *cobalt*, *MASS*, and *pROC* packages. Statistical significance was defined as *p* < 0.05.

## Results

### Study Population

A total of 19,072 patients were screened, of whom 560 underwent endovascular treatment for acute MCA ICAS-LVO. After excluding those treated with rescue strategies other than stenting and cases with missing data on IPTW variables, 264 patients were included in the final analysis: 193 underwent MT + RS and 71 MT only. A flow diagram of patient selection is shown in Supplementary Fig. 1. Among patients treated with MT + RS, self-expandable stents were used in 146/171 procedures (85.4%), whereas balloon-mounted stents were used in 25/171 (14.6%). Peri-procedural antiplatelet regimens were heterogeneous across participating centers, ranging from single-agent therapy to dual antiplatelet therapy and glycoprotein IIb/IIIa inhibitor–based strategies (Supplementary Table 1).

### Variables Associated with Rescue Stenting

Univariate analysis of demographic, baseline, procedural, and angiographic variables associated with receiving RS is reported in Supplementary Table 2. In the multivariable stepwise backward elimination model, variables independently associated with receiving RS were MT failure (defined as inability to achieve mTICI ≥2b after thrombectomy attempts; OR 2.49, 95% CI 1.28–4.90; *p* = 0.01), severe stenosis (> 75%) (OR 2.86, 95% CI 1.45–5.65; *p* = 0.002), and higher baseline Alberta Stroke Program Early CT Score (ASPECTS) (OR 1.29, 95% CI 1.08–1.57; *p* = 0.01) (Table 1). The model showed acceptable discrimination (AUC = 0.75, 95% CI 0.68–0.82) and good calibration according to the Hosmer-Lemeshow test (*p* = 0.12).

**Table 1:** Stepwise multivariable regression analysis for variables associated with receiving RS

variable	OR	95% CI	*p* *
Diabetes	1.82	0.86–4.10	0.12
Baseline ASPECTS	1.29	1.08–1.57	**0.01**
Severe stenosis (> 75%)	2.86	1.45–5.65	**0.002**
Lesion calcification	2.53	0.78–11.41	0.16
MT failure	2.49	1.28–4.90	**0.01**

*RS* rescue stenting, *OR* odds ratio, *CI* confidence interval, *ASPECTS* Alberta Stroke Program Early CT Score, *MT* mechanical thrombectomy, * significance was defined as *p* < 0.05

### Effect Modification Analyses

Having identified MT failure, severe stenosis, and baseline ASPECTS as independent determinants of treatment allocation, we next evaluated whether these same factors modified the association between RS and outcome. Propensity scores were estimated from the treatment-allocation variables, and stabilized IPTW weights achieved adequate balance between treatment groups, with SMDs < 0.10 for all variables (Supplementary Table 3). Propensity score distributions showed adequate overlap, with effective sample sizes remaining close to the original sample sizes across IPTW models (Supplementary Fig. 2). In the overall cohort, the IPTW-weighted proportional-odds model showed no significant association between RS and 90-day clinical outcome compared with MT alone (common OR 0.69, 95% CI 0.40–1.20; *p* = 0.19) (Table 2). Subgroup-specific IPTW-weighted common ORs according to MT failure and stenosis status are reported in Supplementary Table 4. We then evaluated treatment-effect heterogeneity by fitting IPTW-weighted interaction models in which the association between RS and outcome was allowed to vary according to the treatment-allocation variables identified in the first stage of the analysis. In the overall cohort, both MT failure (interaction OR 0.28, 95% CI 0.10–0.83; p for interaction = 0.02) and severe stenosis (interaction OR 0.13, 95% CI 0.03–0.53; p for interaction = 0.004) significantly modified the association between RS and clinical outcome, whereas baseline ASPECTS did not (interaction OR 1.12, 95% CI 0.83–1.54; p for interaction = 0.47) (Table 2).

**Table 2:** IPTW-weighted interaction analyses of the association between rescue stenting and 90-day mRS according to treatment-selection variables

	cOR	iOR	95% CI	*p* *	*p*-int *
IPTW-weighted overall association of RS with 90-day mRS (RS vs. MT only)	0.69	-	0.40–1.20	0.19	-
Overall population interaction analysis (*N* = 264)					
RS × MT failure	-	0.28	0.10–0.83	**-**	**0.02**
RS × Severe stenosis (> 75%)	-	0.13	0.03–0.53	**-**	**0.004**
RS × Baseline ASPECTS ^¶^	-	1.12	0.83–1.54	-	0.47
Context-dependent interaction analyses					
*MT failure subgroup (N = 160)*					
RS × Severe stenosis (>75%)	-	0.25	0.02–3.36	-	0.29
RS × Baseline ASPECTS ^¶^	-	1.02	0.55–1.88	-	0.94
RS × Severe stenosis (>75%) × baseline ASPECTS ^¶^	-	0.08	0.02–0.38	**-**	**0.002**
*Severe stenosis (>75%) subgroup (N = 177)*					
RS × MT failure	-	0.54	0.17–1.76	-	0.31
RS × Baseline ASPECTS ^¶^	-	0.77	0.53–1.12	-	0.17
RS × MT failure × baseline ASPECTS ^¶^	-	0.63	0.25–1.58	-	0.33

*IPTW* inverse probability of treatment weighting, *mRS* modified Rankin Scale, *cOR* common odds ratio, *iOR* interaction odds ratio, *CI* confidence interval, *p-int* p for interaction, *RS* rescue stenting, *MT* mechanical thrombectomy, *ASPECTS* Alberta Stroke Program Early CT Score, *¶* interaction terms represent the effect per 1-point increase in baseline ASPECTS, * significance was defined as *p* < 0.05

**Fig. 1:**
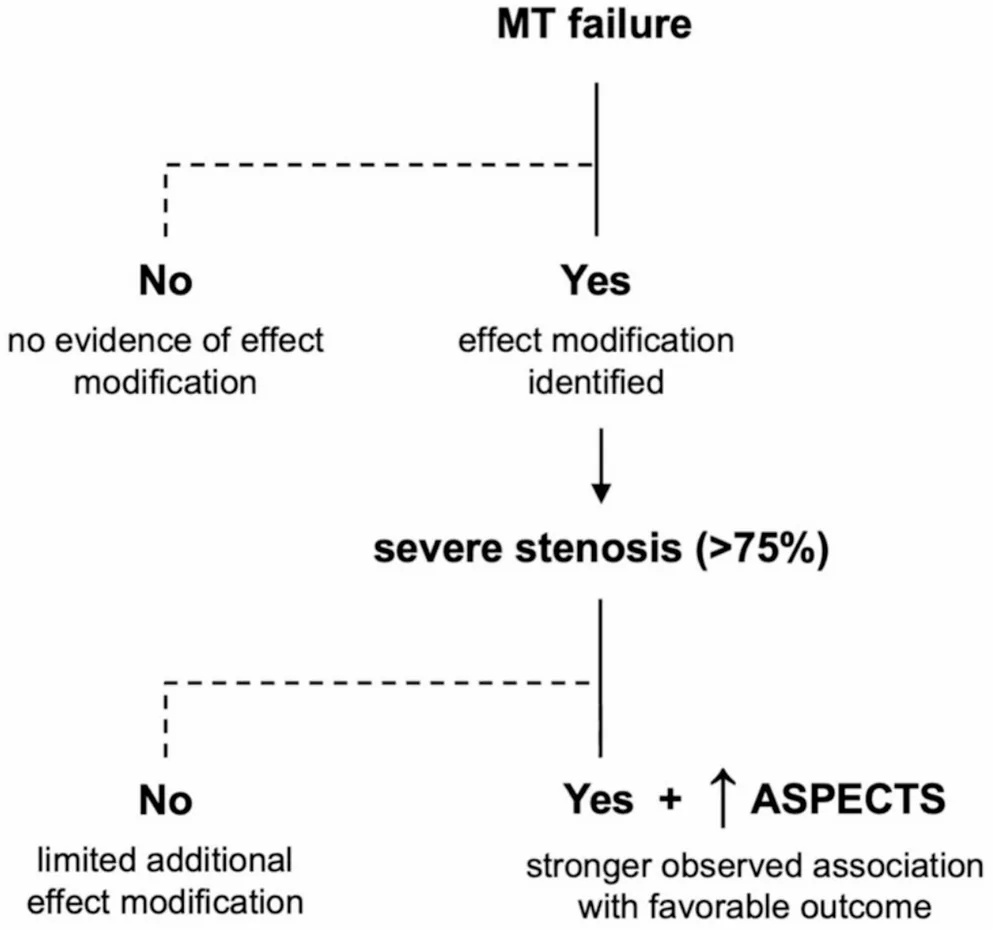
Conceptual summary of the exploratory treatment-effect heterogeneity analyses for rescue stenting in MCA ICAS-LVO. MCA, middle cerebral artery; ICAS, intracranial artery stenosis; LVO, large vessel occlusion; MT, mechanical thrombectomy; RS, rescue stenting; ASPECTS, Alberta Stroke Program Early CT Score

To further explore potential context-specific treatment-effect heterogeneity, we performed subgroup analyses stratified by MT failure and severe stenosis. Within the MT failure subgroup, neither severe stenosis nor baseline ASPECTS alone modified the association between RS and outcome. However, a significant interaction between severe stenosis and baseline ASPECTS was observed (interaction OR 0.08, 95% CI 0.02–0.38; p for interaction = 0.002), suggesting that, among patients with MT failure, the association between RS and functional outcome depended on the combination of stenosis severity and baseline tissue status, with the most favorable association observed in patients with higher baseline ASPECTS. In contrast, within the subgroup of patients with severe stenosis, neither MT failure nor baseline ASPECTS, nor their interaction, significantly modified the association between RS and 90-day mRS score (Table 2). Sensitivity analyses in which the IPTW-weighted outcome models were additionally adjusted for clinically relevant prognostic variables (age, baseline NIHSS score, onset-to-groin time, and intravenous thrombolysis) yielded results consistent with the primary analyses (Supplementary Table 5). A conceptual summary of the exploratory treatment-effect heterogeneity analyses is shown in Fig. 1. 

For angiographic outcomes, neither severe stenosis nor baseline ASPECTS significantly modified the association between RS and final successful recanalization, and no joint interaction was observed. In contrast, baseline ASPECTS significantly modified the association between RS and final complete/near-complete recanalization (interaction OR 1.50, 95% CI 1.06–2.12; p for interaction = 0.02), whereas severe stenosis did not. The association between baseline ASPECTS and complete/near-complete recanalization was similar across stenosis strata, with no evidence that stenosis severity further modified this interaction. For safety outcomes, severe stenosis and ASPECTS showed a possible favorable joint interaction with RS for sICH, although this did not reach statistical significance (interaction OR 0.24, 95% CI 0.05–1.15; p for interaction = 0.08). In contrast, a significant favorable interaction was observed for 90-day mortality (interaction OR 0.12, 95% CI 0.02–0.60; p for interaction = 0.01) (Table 3).

**Table 3:** IPTW-weighted treatment-effect heterogeneity of rescue stenting for secondary outcomes according to severe stenosis and baseline ASPECTS in patients with MT failure

Outcome measure/variable	iOR	95% CI	*p*-int *
final successful recanalization (mTICI 2b-3)			
RS × Severe stenosis (> 75%)	4.01	0.96–16.90	0.06
RS × Baseline ASPECTS ^¶^	1.10	0.76–1.61	0.60
RS × Severe stenosis (>75%) × baseline ASPECTS ^¶^	1.95	0.69–5.50	0.21
final complete/near-complete recanalization (mTICI 2c-3)			
RS × Severe stenosis (>75%)	2.62	0.77–8.89	0.12
RS × Baseline ASPECTS ^¶^	1.50	1.06–2.12	**0.02**
RS × Severe stenosis (>75%) × baseline ASPECTS ^¶^	0.94	0.34–2.59	0.90
sICH			
RS × Severe stenosis (> 75%)	0.80	0.17–3.79	0.78
RS × Baseline ASPECTS ^¶^	1.08	0.63–1.85	0.78
RS × Severe stenosis (>75%) × baseline ASPECTS ^¶^	0.24	0.05–1.15	0.08
90-day mortality			
RS × Severe stenosis (> 75%)	0.55	0.04–8.34	0.67
RS × Baseline ASPECTS ^¶^	0.96	0.53–1.74	0.89
RS × Severe stenosis (>75%) × baseline ASPECTS ^¶^	0.12	0.02–0.60	**0.01**

*IPTW* inverse probability of treatment weighting, *ASPECTS* Alberta Stroke Program Early CT Score, *MT* mechanical thrombectomy, *iOR* interaction odds ratio, *CI* confidence interval,* p-int* p for interaction, *mTICI* modified treatment in cerebral infarction, *RS* rescue stenting, *sICH* symptomatic intracerebral hemorrhage, ¶ interaction terms represent the effect per 1-point increase in baseline ASPECTS, * significance was defined as *p* < 0.05

## Discussion

In this multicenter retrospective cohort of patients with acute MCA ICAS-LVO, we found that the decision to perform RS was primarily associated with MT failure, severe residual stenosis (> 75%), and higher baseline ASPECTS. The association between MT failure and the decision to perform stenting is consistent with current clinical practice, in which RS is typically considered when thrombectomy alone fails to achieve durable recanalization [13–15]. In the overall IPTW-weighted analysis, using propensity scores derived from the treatment allocation variables identified in the first stage of the analysis, RS was not significantly associated with 90-day functional outcome compared with MT alone. However, the association between RS and functional outcome differed according to both MT failure and severe stenosis. When the analysis was restricted to patients with MT failure, treatment-effect heterogeneity appeared to depend on the combined influence of severe stenosis and ASPECTS rather than on either factor alone. In this subgroup, the association between RS and both functional outcome and survival was more favorable in patients with severe stenosis and preserved baseline imaging, alongside a parallel, albeit non-significant, favorable association with sICH. By contrast, conditioning the RS analysis first on severe stenosis did not further identify meaningful treatment-effect heterogeneity.

Prior observational studies and meta-analyses have suggested that rescue therapies in ICAS-LVO are associated with improved functional outcomes and lower mortality compared with conservative management, without a significant increase in sICH [5, 11, 20]. Nonetheless, there remains marked heterogeneity in the management of unexpected ICAS during MT, underscoring how often the decision to stent is driven by intraprocedural findings rather than standardized evidence [13–15]. Against this background, the absence of a significant overall association between RS and outcome, together with the observed treatment-effect heterogeneity, suggests that the potential value of RS may depend on the clinical context in which it is performed. Specifically, MT failure may represent the context in which lesion severity and baseline tissue status jointly differentiate the association between RS and outcome, with the most favorable association observed in patients with severe stenosis and preserved baseline ASPECTS. One possible interpretation is that severe stenosis likely reflects a lesion in which the atherosclerotic substrate is the primary mechanism of thrombectomy failure and recurrent instability, making definitive luminal scaffolding more therapeutically relevant [21, 22]. At the same time, higher ASPECTS modified the association between RS and complete/near-complete recanalization in a favorable direction, suggesting that the clinical pattern observed in patients with favorable baseline imaging may be partly mediated by improved technical effectiveness rather than baseline prognosis alone. This interpretation is also consistent with prior studies suggesting that preserved ASPECTS may identify patients more likely to benefit from successful reperfusion strategies [23]. Although exploratory, this pattern may reflect both biological and procedural factors, including reduced microvascular injury [24] and greater operator persistence in patients with smaller infarct cores.

These considerations may help reconcile discrepancies between retrospective series and a recent randomized comparison of angioplasty with or without stenting versus no rescue endovascular treatment, which failed to show a clear overall benefit of rescue strategies in patients with unsuccessful recanalization or severe residual stenosis after MT [5, 12]. One possible explanation is that a broader eligibility based solely on MT failure or severe fixed residual stenosis may have contributed to an attenuation of treatment effects across biologically distinct subgroups [12]. Our findings suggest that future prospective studies may benefit from moving beyond a purely binary recanalization-based framework and incorporating both hemodynamically relevant stenosis and pretreatment infarct burden when selecting candidates for RS. In this context, additional insights will likely be provided by the ongoing Permanent Intracranial STenting for Acute Refractory large vessel occlusions (PISTAR; NCT06071091) and the IntraCranial Atherosclerosis Related Large-vessel Occlusion Treated With Urgent Stenting (ICARUS; NCT06472336) randomized clinical trials [25].

Several limitations should be acknowledged. First, the retrospective observational design introduces the possibility of residual confounding and treatment-by-indication bias. The propensity-score models were based on variables independently associated with real-world RS treatment allocation, in accordance with the study objective, rather than on a broader set of recorded variables. Although the main findings remained consistent after additional adjustment for age, baseline NIHSS, onset-to-groin time, and intravenous thrombolysis, unmeasured clinical features or nuanced angiographic factors, not fully captured in our registry, may still have influenced both treatment allocation and outcomes. Second, the absence of a centralized core laboratory for angiographic review introduces the potential for measurement variability. In addition, residual stenosis was assessed angiographically after restoration of antegrade flow following thrombectomy. Because vessel caliber and lesion appearance may change after clot retrieval, some variability in residual stenosis estimation cannot be excluded. Third, heterogeneity in endovascular techniques, stent devices, and peri-procedural antiplatelet management across participating centers represents an additional source of variability. Although these procedural characteristics were collected and are reported descriptively, the predominance of self-expandable stents and the diversity of antiplatelet regimens precluded meaningful exploratory analyses according to device class or pharmacological strategy. Fourth, subgroup analyses, particularly those exploring higher-order interactions, should be interpreted cautiously because subgroup sample sizes were limited and these analyses were exploratory and hypothesis-generating. Moreover, the common odds ratios derived from ordinal logistic regression assume proportional odds across mRS thresholds, which may not hold uniformly in all interaction models. Fifth, our cohort predominantly included patients with relatively favorable baseline ASPECTS, which may limit the applicability of our findings to patients with larger infarct cores, in whom the balance between benefit and risk of RS may differ. The limited representation of patients with lower ASPECTS also precluded robust evaluation of treatment-effect heterogeneity across clinically relevant ASPECTS categories. Finally, the present cohort was restricted to MCA ICAS-LVO, which improves homogeneity but may limit extrapolation to other vascular territories.

## Conclusions

In patients with MCA ICAS-LVO, MT failure, severe residual stenosis, and favorable baseline imaging characterize the real-world allocation of RS. Within this observational cohort, RS appeared to be associated with more favorable functional outcomes among patients with MT failure, particularly when severe residual stenosis coexisted with preserved ASPECTS. These findings support the concept that treatment-effect heterogeneity in ICAS-related LVO extends beyond recanalization status alone and is influenced by both lesion-specific and tissue-level imaging characteristics. However, these observations remain hypothesis-generating and require prospective validation before being incorporated into standardized treatment algorithms.

## Supplementary Information

Below is the link to the electronic supplementary material.

Supplementary Material 1 (PDF 895 KB)

## Data Availability

No datasets were generated or analysed during the current study.
