# Supplementary material for: Effect Modification of Rescue Stenting in ICAS-Related Middle Cerebral Artery Occlusion: The Role of Thrombectomy Failure, Stenosis Severity, and ASPECTS
Source: Transl Stroke Res. 2026 Sep 23;17(5):105. doi: 10.1007/s12975-026-01496-9 (PMC13597473; doi:10.1007/s12975-026-01496-9)
Supplement: Supplementary file 1 [file 12975_2026_1496_MOESM1_ESM.pdf]

Effect modification of rescue stenting in ICAS-related middle cerebral artery occlusion: the role of thrombectomy failure, stenosis severity, and ASPECTS

Supplementary Material

Supplementary Figure 1. Flow diagram of patient selection

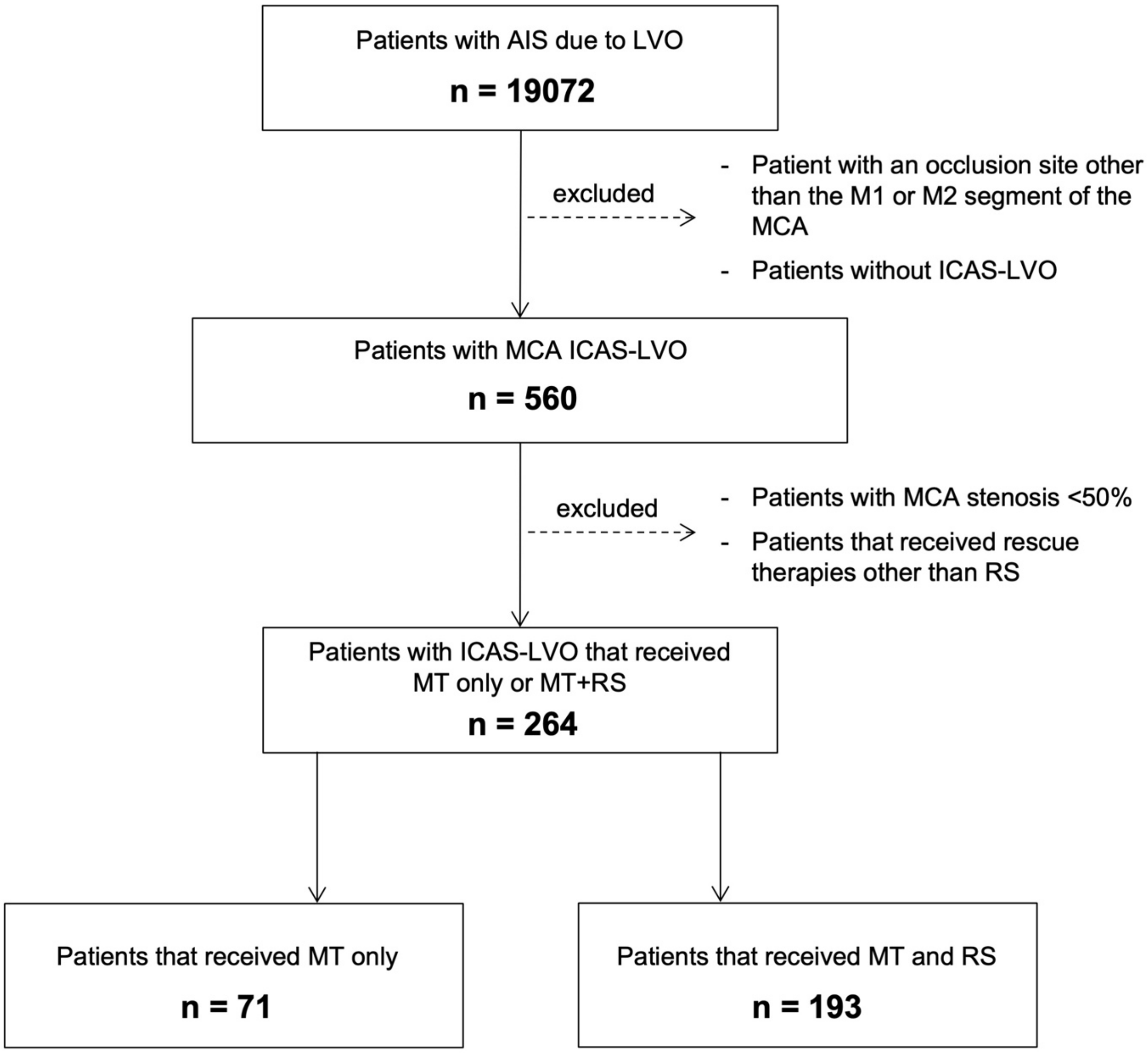

AIS, acute ischemic stroke; LVO, large vessel occlusion; MCA, middle cerebral artery; ICAS, intracranial artery stenosis; RS, rescue stenting; MT, mechanical thrombectomy.

Supplementary Table 1. Other procedural features of rescue stenting

**Stent type, n/N (%)**	
Self-expanding stent for flexible aneurysm treatment	
ACCLINO stent® (Acandis)	30/171 (17.5)
Neuroform Atlas® Stent System (Stryker)	37/171 (21.6)
ENTERPRISE™ Vascular Reconstruction Device (J&J)	10/171 (5.8)
pEGASUS Stent System (Phenox)	6/171 (3.5)
LVIS stent (Terumo)	3/171 (1.8)
Self-expanding stent for intracranial artery stenosis treatment	
CREDO® Stent (Acandis)	20/171 (11.7)
Wingspan Stent System (Stryker)	5/171 (2.9)
Detachable self-expanding stent for mechanical thrombectomy	
Solitaire AB™ (Medtronic)	35/171 (20.5)
Balloon-mounted coronary stents	
Pro-Kinetic Energy stent (Biotronik)	3/171 (1.8)
Resolute Onyx™ stent (Medtronic)	20/171 (11.7)
Resolute Integrity™ stent (Medtronic)	1/171 (0.6)
Sinergy™ stent (Boston Scientific)	1/171 (0.6)
**Type of peri-procedural antiplatelets agent, n/N (%)**	
aspirin	21/193 (10.9)
cangrelor	15/193 (7.8)
GPI	74/193 (38.3)
clopidogrel	4/193 (2.1)
aspirin + clopidogrel	18/193 (9.3)
aspirin + GPI	28/193 (14.5)
aspirin + clopidogrel + GPI	10/193 (5.2)
clopidogrel + GPI	3/193 (1.6)
aspirin + cangrelor	7/193 (3.6)

GPI, glycoprotein IIb/IIIa inhibitors

Supplementary Table 2. Demographic, baseline, procedural, angiographic, and unadjusted clinical outcome characteristics according to rescue stenting status.

	MT-only (N = 71)	MT+RS (N = 193)	p*
**Demographics and baseline characteristics**			
Age, median (IQR)	72 (63-79)	69 (59-77)	0.08
Sex (female), n/N (%)	34/71 (47.9)	72/193 (37.3)	0.12
Hypertension, n/N (%)	46/71 (64.8)	144/193 (74.6)	0.12
Dyslipidemia, n/N (%)	27/71 (38.0)	83/193 (43.0)	0.47
Diabetes, n/N (%)	13/71 (18.3)	61/193 (31.6)	**0.03**
Atrial fibrillation, n/N (%)	17/71 (23.9)	30/193 (15.5)	0.11
Coronary artery disease, n/N (%)	7/71 (10.0)	30/193 (15.7)	0.24
Prior stroke, n/N (%)	10/71 (14.1)	37/193 (19.2)	0.34
Prior antiplatelets, n/N (%)	12/68 (17.6)	44/193 (22.8)	0.37
Prior anticoagulants, n/N (%)	9/69 (13.0)	25/193 (12.9)	0.99
Pre-event mRS score, median (IQR)	0 (0-1)	0 (0-0)	0.84
NIHSS score, median (IQR)	12 (7-17)	12 (7-18)	0.78
Left-side stroke, n/N (%)	39/71 (54.9)	108/193 (55.9)	0.95
M1-MCA segment occlusion, n/N (%)	60/71 (84.5)	163/193 (84.5)	1.00
baseline ASPECTS, median (IQR)	8 (6-9)	9 (8-10)	**<0.001**
**Procedural and angiographic data**			
IVT, n/N (%)	20/71 (28.2)	52/193 (26.9)	0.84
Onset-to-groin (minutes), median (IQR)	340 (240-514)	300 (200-490)	0.60
General anesthesia, n/N (%)	41/71 (57.1)	118/193 (61.1)	0.62
Lesion length (mm), median (IQR)	8 (5-11)	8 (5-10)	0.07
Lesion type according to Mori classification, n/N (%)			0.89
Type A	17/68 (25.0)	54/193 (28.0)	
Type B	32/68 (47.1)	87/193 (45.1)	
Type C	19/68 (27.9)	52/193 (26.9)	
Lesion calcification, n/N (%)	3/69 (4.3)	27/191 (14.3)	**0.02**
Severe stenosis (>75%), n/N (%)	35/71 (49.3)	142/193 (73.6)	**<0.001**
Number of passes, median (IQR)	2 (1-3)	2 (1-3)	0.34
MT failure, n/N (%)	27/71 (38.0)	133/193 (68.9)	**<0.001**
**90-day mRS score, n/N (%)**			
0	12/71 (16.9)	23/193 (11.9)	
1	6/71 (8.5)	34/193 (17.6)	
2	12/71 (16.9)	33/193 (17.1)	
3	5/71 (7.0)	35/193 (18.1)	
4	14/71 (19.7)	30/193 (15.6)	
5	10/71 (14.1)	12/193 (6.2)	
6	12/71 (16.9)	26/193 (13.5)	

MT, mechanical thrombectomy; RS, rescue stenting; IQR, interquartile range; mRS, modified Rankin scale; NIHSS, National Institutes of Health Stroke Scale; MCA, middle cerebral artery; ASPECTS, Alberta Stroke Program Early CT score; IVT, intravenous thrombolysis; * significance was defined as $p < 0.05$.

Supplementary Table 3. Covariate balance before and after IPTW

	standardized mean difference	
	**before IPTW**	**after IPTW**
**Overall population analysis**		
MT failure	0.651	0.014
Severe stenosis (>75%)	0.515	- 0.018
baseline ASPECTS	0.484	0.071
**Context-dependent interaction analyses**		
MT failure subgroup		
Severe stenosis (>75%)	0.043	0.039
baseline ASPECTS	0.543	0.093
Severe stenosis (>75%) subgroup		
MT failure	0.295	0.054
baseline ASPECTS	0.466	0.025

IPTW, inverse probability of treatment weighting; MT, mechanical thrombectomy; ASPECTS, Alberta Stroke Program Early CT Score.

Supplementary Figure 2. Propensity score overlap and IPTW diagnostics for the overall cohort, MT failure subgroup, and severe stenosis subgroup.

Overall population

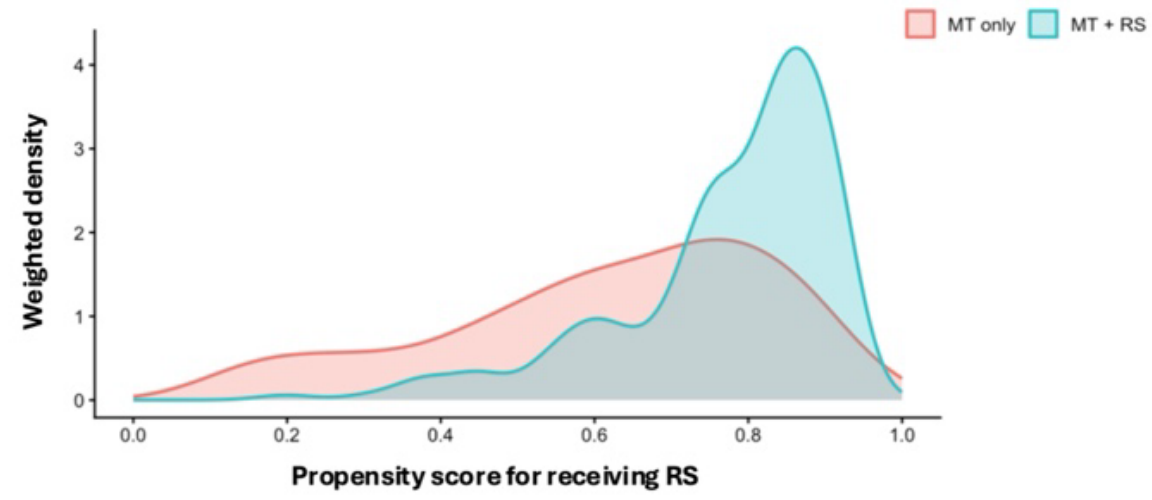

Treatment type	Original sample size	Effective sample size	Median IPTW weight	Weight range
MT only	71	50.9	0.73	0.32-3.01
MT + RS	193	176.0	0.88	0.80-3.68

MT failure subgroup

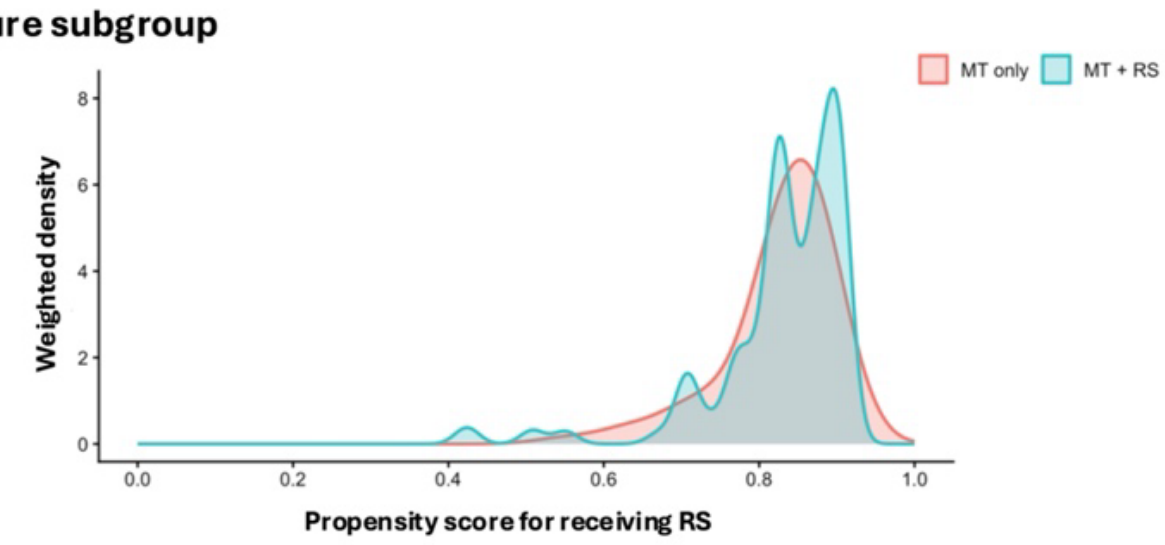

Treatment type	Original sample size	Effective sample size	Median IPTW weight	Weight range
MT only	27	24.0	0.97	0.38-1.76
MT + RS	133	130.8	0.98	0.92-1.21

Severe stenosis subgroup

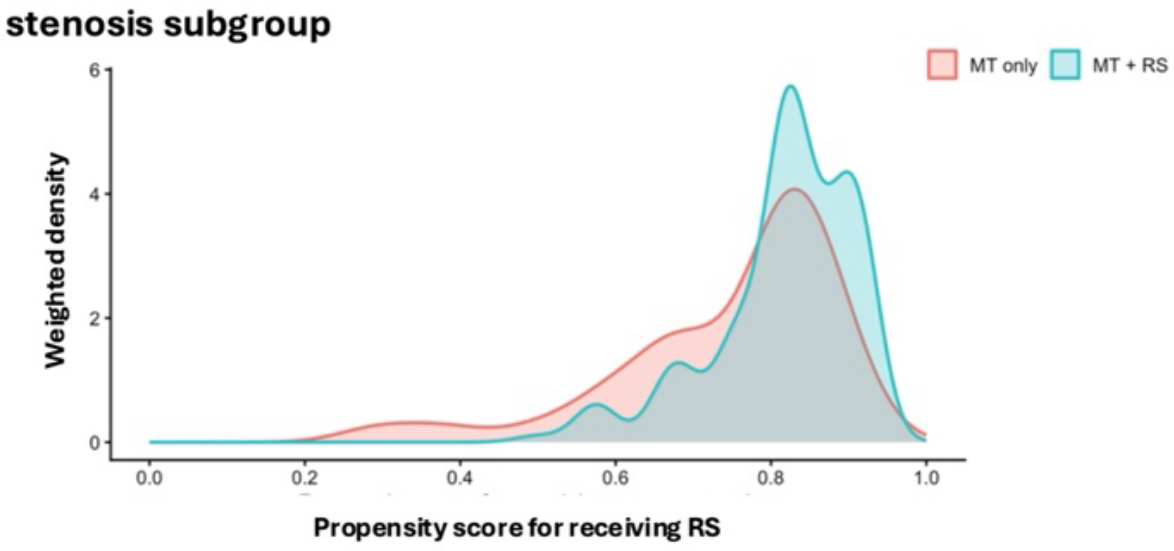

Treatment type	Original sample size	Effective sample size	Median IPTW weight	Weight range
MT only	35	29.0	1.07	0.28-2.28
MT + RS	142	139.6	0.97	0.88-1.61

Density plots illustrate the distribution of estimated propensity scores according to treatment group. Tables report original sample size, effective sample size (ESS), median stabilized weight, and weight range. ESS values close to the original sample size indicate stable weighting with limited loss of precision. IPTW, inverse probability of treatment weighting; MT, mechanical thrombectomy; RS, rescue stenting.

Supplementary Table 4. IPTW-weighted subgroup-specific associations between rescue stenting and 90-day mRS according to MT failure and stenosis status.

Subgroup	cOR	95% CI	p *
MT failure	0.40	0.18 - 0.90	**0.03**
MT success	1.48	0.72 - 3.05	0.28
Stenosis severity >75%	0.39	0.21 - 0.73	**0.003**
Stenosis severity <75%	2.82	0.86 - 9.25	0.08

IPTW, inverse probability of treatment weighting; mRS, modified Rankin Scale; MT, mechanical thrombectomy; cOR, common odds ratio; CI, confidence interval; * significance was defined as $p < 0.05$.

Supplementary Table 5. Sensitivity analysis of IPTW-weighted interaction models for 90-day mRS score after additional adjustment for age, baseline NIHSS score, IVT and onset-to-groin time, according to treatment-selection variables.

	cOR	iOR	95% CI	p *	p-int *
**IPTW-weighted overall association of RS with 90-day mRS (RS vs. MT only)**	0.54	-	0.29 - 1.01	0.05	-
**Overall population interaction analysis (N=233)**					
RS × MT failure	-	0.20	0.06 - 0.66	**-**	**0.008**
RS × Severe stenosis (>75%)	-	0.10	0.03 - 0.38	**-**	**0.001**
RS × Baseline ASPECTS ¶	-	1.10	0.75 - 1.60	-	0.64
**Context-dependent interaction analyses**					
MT failure subgroup (N=141)					
RS × Severe stenosis (>75%)	-	0.11	0.01 - 1.05	-	0.05
RS × Baseline ASPECTS ¶	-	1.06	0.50 - 2.24	-	0.88
RS × Severe stenosis (>75%) × baseline ASPECTS ¶	-	0.05	0.01 - 0.18	**-**	**<0.001**
Severe stenosis (>75%) subgroup (N=154)					
RS × MT failure	-	0.34	0.09 - 1.29	-	0.11
RS × Baseline ASPECTS ¶	-	0.75	0.48 - 1.19	-	0.22
RS × MT failure × baseline ASPECTS ¶	-	0.54	0.20 - 1.46	-	0.22

IPTW, inverse probability of treatment weighting; mRS, modified Rankin Scale; NIHSS, National Institutes of Health Stroke Scale; IVT, intravenous thrombolysis; cOR, common odds ratio; iOR, interaction odds ratio; CI, confidence interval; p-int, p for interaction; RS, rescue stenting; MT, mechanical thrombectomy; ASPECTS, Alberta Stroke Program Early CT Score; ¶, interaction terms represent the effect per 1-point increase in baseline ASPECTS; * significance was defined as $p < 0.05$.
